# Shared manufacturing service evaluation based on intuitionistic fuzzy VIKOR

**DOI:** 10.1016/j.heliyon.2024.e29250

**Published:** 2024-04-09

**Authors:** Jiating Liang, Peng Liu

**Affiliations:** School of Management, Shenyang University of Technology, Shenyang, 110870, China

**Keywords:** Shared manufacturing, Service evaluation, Intuitionistic fuzzy sets, VIKOR method

## Abstract

With the rise of the concept of sharing economy, the shared manufacturing model has gained widespread attention. The VIKOR shared manufacturing service evaluation approach is presented based on an intuitionistic fuzzy environment, which enables users to filter out acceptable shared manufacturing services from a wide pool of shared manufacturing services with similar functional qualities. Firstly, considering the QOS multi-indicator comprehensive evaluation of services by multiple stakeholders under the fundamental characteristics of shared manufacturing, the QOS evaluation index system is built from the two aspects of online and offline, which includes 2 first-level indicators and 10 second-level indicators. The paper also constructs a service recommendation model considering supply and demand constraints. Secondly, the intuitionistic fuzzy numbers are introduced to define the non-quantitative indexes, and the G1-method and variable-precision rough set theory are used for the assignment, and the maximum entropy theory is utilized to integrate the assignment method to obtain the combination weights. Thirdly, the VIKOR method based on intuitionistic fuzzy sets is used to evaluate and rank the shared manufacturing candidate services, in which the combined benefits and minimum regret values of the groups are solved based on the intuitionistic fuzzy number similarity. Finally, the reliability and feasibility of the algorithm are verified with a real case.

## 1 Introduction

With the rise of the "sharing economy" as a new economic model, the manufacturing industry from China and the global has ushered in new opportunities. The effective combination of the sharing economy and the manufacturing industry has led to the emergence of the shared manufacturing model. Shared manufacturing is a new model of the manufacturing industry based on the concept of sharing, which gathers the idle and dispersed resources of each manufacturing enterprise, dynamically and efficiently sharing the aggregated resources to the demand side through flexible matching. In fact, the concept of shared manufacturing was first proposed by Ellen Brandt [1] in 1990, and some sizable companies overseas have already implemented it. For example, MFG, the largest trading platform created in the United States in 2000, GE's Predix, Siemens' Mindsphere, Uber, Airbnb and others are developing fast. Yu et al. [2] study the concept, definition and service operations of shared manufacturing in the sharing economy. He et al. [3] consider the shared manufacturing in Chinese manufacturing industry. The shared manufacturing model has gained traction in our manufacturing industry in recent years due to the rapid development of advanced technologies like cloud computing, internet of things, and physical information systems, such as the “Taobao Factory” in Alibaba, Haier's Hai Chuang Foreign Exchange, Shenyang Machine Tool. With the traditional Web manufacturing, cloud manufacturing model, it has become the current hot research of our scholars. On the shared manufacturing platform, it concentrates plentiful shared manufacturing services with similar functional attributes but vastly different non-functional attributes. Since direct combination optimization will affect the efficiency of optimization, it is imperative to assess the shared manufacturing services chosen initially as this sets the stage for the subsequent service combination optimization process. It is an important task to evaluate many shared manufacturing services with similar function attributes and select the best service to form the best shared manufacturing service composition.

Numerous national and international studies are currently being conducted on the assessment and optimization of shared manufacturing service portfolios. Chen et al. [4] proposed a differentiated duopoly model considering capacity constraints and shared manufacturing. Xie et al. [5] present an optimal or near-optimal matching algorithm to cope with a large volume of capacity-sharing problems. Zhang et al. [6] proposed hybrid sensing-based approach for the monitoring and maintenance of shared manufacturing resources. Yan et al. [7] reviewed the research on supply-demand matching and scheduling of shared manufacturing platform from the perspective of sharing economy and platform operation. Jiang et al. [8] studied the choice of shared manufacturing model and decision-making optimization under the competition-cooperation relationship, constructed a game model, and gave the optimal selection strategy of supply and demand sides. Cao et al. [9] constructed a capacity splitting and matching model for the capacity matching problem on the shared manufacturing platform with the goal of maximizing the revenue of the platform. Pu et al. [10] addressed the problem of stable matching between supply and demand on a shared manufacturing platform and constructed a stable matching optimization model with the objective of maximizing the satisfaction of both supply and demand and the revenue of the platform. Guo and Dong [11] constructed an ability matching optimization model for shared manufacturing platform with the objectives of on-time rate, production quality and transportation cost, and proposed an improved multi-objective particle swarm optimization algorithm to solve the model. Zhang et al. [12] was aiming at the problems of service providers on third-party shared platforms, constructed a dynamic supply chain model with platform as the leading factor, and discussed the competition intensity among service providers, and then the optimal decision of supply chain system was further investigated. Veerababu Ramakurthia et al. [13] developed a new multi-objective-based Hybridized Moth Flame Evolutionary Optimization Algorithm (HMFEO) for resource sharing and scheduling in a sustainable distributed manufacturing system. Liu and Liu [14] described the dynamic allocation of tasks and resources in shared manufacturing environment and proposed non-dominated sorting genetic algorithm (NSGA-II) and multi-objective particle swarm optimization (MOPSO) to solve the model. Hao and Wang [15] had built a tripartite evolutionary game model of firm A, firm B and consumers, and based on the principle of System dynamics, the evolutionary game process was dynamically simulated using MATLAB. Wang et al. [16] built a digital dual-drive service model to realize seamless monitoring of shared manufacturing resources and built a resource allocation model based on considering the credit of resource providers.

For service evaluation methods, multi-objective decision-making is the most widely used service evaluation method [17], such as the common TOPSIS evaluation method, and DEA evaluation method, grey correlation method and comprehensive evaluation of extension [18]. Hu et al. [19] adopted a combination of qualitative and quantitative methods based on fuzzy comprehensive evaluation (FCE) and established a comprehensive evaluation index system and fuzzy trapezoidal membership function. Yang et al. [20] proposed a service satisfaction-based trust evaluation model for cloud manufacturing. Sun et al. [21] proposed an evaluation framework for IT-enabled service-oriented manufacturing. These kinds of approaches, however, are not adaptable enough to handle intricate fuzzy multi-objective decision-making scenarios. The VIKOR method (Vlsekriterijumska Optimizacija I Kompromisno Resenje multi-criteria compromise solution ranking method) obtains the compromise solution closest to the ideal solution, that is, the compromise solution with priority, it makes the maximum combination benefits and the minimum regret values more acceptable and has the characteristics of being able to deal with conflict attributes. Cheng and Yu [22] proposed a service selection method considering user preferences and used VIKOR to evaluate candidate services in an intuitionistic fuzzy environment. Liang et al. [23] proposed a method to evaluate candidate services by combining multilevel hybrid fuzzy best-worst method with VIKOR. Fu and Zhou [24] studied the selection of energy service companies based on intuitionistic fuzzy entropy and VIKOR framework, which provided a new idea for multi-attribute intuitionistic fuzzy group decision-making. Delaram et al. [25] studied a matching mechanism for public cloud manufacturing platforms using intuitionistic Fuzzy VIKOR and deferred acceptance algorithm. Rodriguez et al. [26] used fuzzy constraint AHP and TOPSIS integrated method to evaluate and select equipment suppliers. Rakesh D. Raut et al. [27] proposed a DEA and Artificial neural network Method to evaluate and select suppliers. Ma and Chen [28] proposed a method based on interval and grey correlation degree for manufacturing service resources comprehensive matching recommendation. In this process, the quality-of-service QOS (Quality of Service) is the key evaluation index to screen out the candidate services to meet the needs of users. Therefore, the research focus of this paper is how to establish a good, shared manufacturing quality of service evaluation index system, and empower this evaluation system indicators, and finally based on the index system to build a reasonable service recommendation model.

In this paper, VIKOR evaluation method based on intuitionistic fuzzy set is used to rank the candidate services in shared manufacturing environment to help users select satisfactory service providers. The main innovations of this paper are as follows: In dealing with uncertain information, this paper adopts IFS (intuitionistic fuzzy set) method, which considers hesitancy degree, it is more flexible and practical than traditional fuzzy sets; In the aspect of determining the index weight, this paper adopts combined assignment method---G 1-method and variable precision rough set theory method, it can solve the problems that the traditional comprehensive weighting method is not reliable because of its dependence on subjectivity and cannot play the role of subjective and objective weighting methods; In the evaluation method, this paper adopts VIKOR method. Compared with other evaluation methods, it makes the decision-making result more reasonable and dependable and has the characteristics of dealing with the conflict attribute.

The remainder of this paper is organized as follows. Section 2 presents the problem descriptions and QOS evaluation index system. Section 3 provides the model constructions and solutions. Section 4 provides numerical analysis. Section 5 concludes the paper.

## 2 Problem description and QOS evaluation index system

### 2.1 Description of the problem

**Fig. 1:**
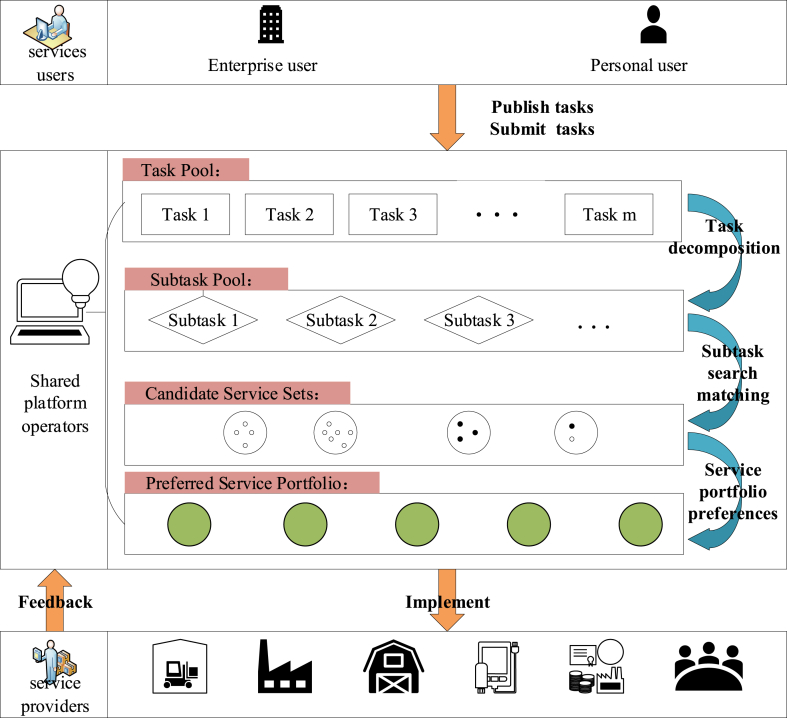
Shared manufacturing platform operation diagram.

The shared manufacturing platform operates as shown in Fig. 1 below. In Fig. 1, shared manufacturing consists of three parts: users who need services, service providers, and third-party platforms. In the sharing environment, the service provider will virtualize the idle resources, including equipment, sites, materials, through the shared platform and encapsulate these resources in the shared platform; After the user places a demand, the platform breaks down the task requirements, looks for and matches subtasks on the shared platform, and provides high-quality services to satisfy the user's needs; finally, one or more service providers complete the tasks of the user.

**Fig. 2:**
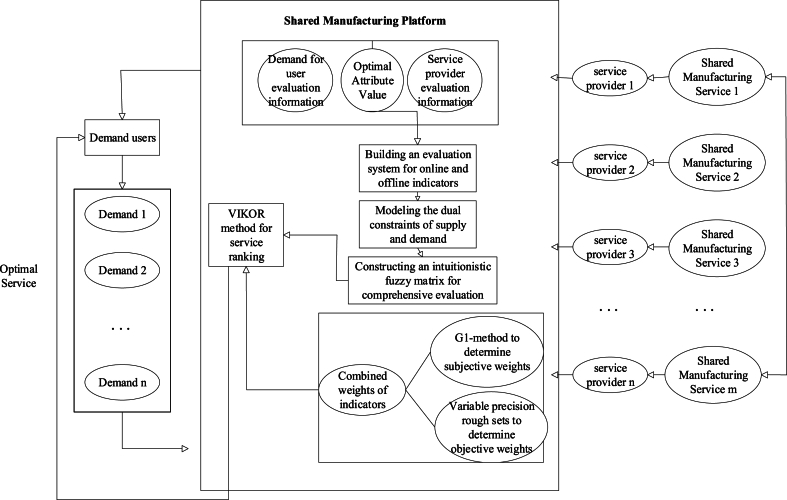
QOS model for shared manufacturing services.

In this paper, the QOS model shown in Fig. 2 is constructed. The shared manufacturing platform, service providers, and demand users are the three segments of the model. The services provided by the service providers are encapsulated in the shared platform, and when the user puts forward the task demand, it submits the demand application to the platform, and the platform searches the encapsulated services based on the demand; then, the shared platform sorts and optimizes the shared manufacturing services from the QOS indexes of the demand user and the service providers, and feeds back the final sorting results to the demand user, and the users evaluate the optimal service recommended by the platform, and their evaluation results will become more and more realistic as the number of times the shared manufacturing service runs increases.

### 2.2 QOS evaluation indicator system

**Fig. 3:**
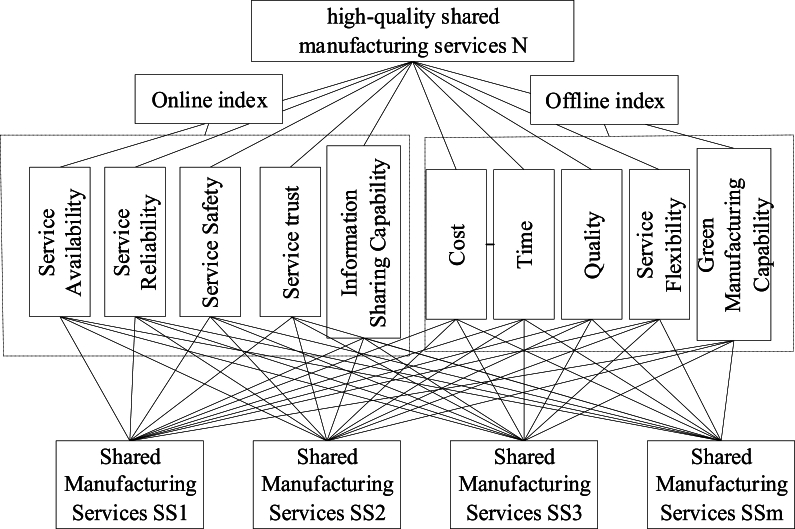
Shared manufacturing service evaluation system.

Shared Manufacturing Services (SMS) includes online transactions and offline production. When it comes to meeting the demands of online transactions, shared manufacturing must prioritize the internet more than traditional manufacturing services. Conversely, when it comes to Web cloud services, shared manufacturing must prioritize offline production capabilities. Therefore, according to comprehensiveness, accuracy, applicability and simplicity, this paper constructs a QOS evaluation index system based on on-line and off-line, including 2 first-level indexes and 10 second-level indexes. The shared manufacturing service evaluation system is shown as Fig. 3.

#### 2.2.1 Online indicators

Like Web cloud services, the online portion of shared manufacturing services demands strong network service capabilities. This study presents new online assessment indicators based on the features of the online shared manufacturing transaction, primarily on the widely used QOS evaluation indexes.

(1) Service availability $\left(S_{1}\right)$: the ratio of shared manufacturing service downtime to invoked time.
(2) Service reliability $\left(S_{2}\right)$: the ratio of the number of times a shared manufacturing service successfully responds to demand to the number of times it is called.
(3) Service safety $\left(S_{3}\right)$: the ability of the service provider to protect and encrypt user data transmission.
(4) Service trust $\left(S_{4}\right)$: the overall evaluation of the service provider of task completion by users.
(5) Information Sharing Capability $\left(S_{5}\right)$: the timeliness and completeness of the service providers' ability to share its manufacturing and service information in the platform with other service providers working together.

#### 2.2.2 Offline indicators

The off-line production portion of shared manufacturing services must guarantee that the services are provided in a fair amount of time and at a reasonable cost, and that the items are delivered in compliance with all applicable regulations and standards of quality. Because there is unpredictability throughout the entire service process for a complicated manufacturing assignment, service flexibility is crucial for shared manufacturing services. Green manufacturing capacity—which considers environmental concerns like carbon dioxide emissions—also needs to be taken into account. The following are the primary offline indexes that this paper has chosen.

(1) Cost $\left(S_{6}\right)$: the cost paid by the user to acquire the shared manufacturing service, such as processing cost, delivery cost.
(2) Time $\left(S_{7}\right)$: the time interval between the user's service request and the service providers' response to the service.
(3) Quality $\left(S_{8}\right)$: the qualification rate of the service provided by the service provider and the user's satisfaction with the service.
(4) Service Flexibility $\left(S_{9}\right)$: the service providers' ability to cope with uncertainties (changes in production orders, changes in manufacturing capacity).
(5) Green Manufacturing Capability $\left(S_{10}\right)$: the service providers' capability of green manufacturing in the process of service provision.

### 2.3 A system of double constraints on supply and demand

#### 2.3.1 Demand user constraint indicators

The service evaluation indexes reflect the non-functional requirements of the users and take the indexes in the evaluation system as constraints to form the constraint conditions of the users. For negative indicators, it should be less than or equal to the maximum acceptable value, such as time, cost. For positive indicators, it should be greater than or equal to the minimum acceptable value, such as service reliability, service security. The specific definitions are as follows:

$$q_{ns}\leq q_{s}^{\max},s=c、t$$

$$q_{ns}\geq q_{s}^{\min},s=sav、sre、ssa、scre、insc、qua、\text{sfle}、\text{grca}$$

where $q_{s}^{\max}$ denotes the maximum receivable value of the demand user for the QOS attribute and $q_{s}^{\min}$ denotes the minimum receivable value of the demand user for the QOS attribute.

#### 2.3.2 Service provider constraint indicators

The service provider provides services to the users through the shared platform. If the constraint indexes of the users fail to meet the requirements of the service provider, the service provider refuses to provide services for the user. From the point of view of service providers, this paper puts forward demand user payment speed and demand user credibility as constraint indicators.

(1) Demand User Payment Speed (dps)

Demand user payment speed refers to the speed at which the demand user pays money to the service provider. The sharing platform takes the historical average payment time of the demand user as its payment speed, the service provider determines the range of acceptable payment speed, and the platform recommends the services within this range, $dps\geq dps_{n}^{\min}$, $dps_{n}^{\min}$ is the slowest payment speed acceptable to the service provider.

(2) Demand User Credibility (dcy)

Demand user credibility refers to the evaluation value of credibility given by the shared platform based on the demand user's own conditions, such as registered capital, years of experience, credit status et al. The higher the credibility of the demand user, the better the service provider's credibility will be. The higher the credibility of the demand user, the more willing the service provider is to provide services. The service provider gives an acceptable range within which the platform recommends services, $dcy\geq dcy_{n}^{\min}$, $dcy_{n}^{\min}$ is the minimum credibility of the demand user acceptable to the service provider.

## 3 Comprehensive evaluation modeling of shared manufacturing services

### 3.1 Heterogeneous QOS data processing

(1) intuitionistic fuzzy set theory

Compared to traditional decision-making methods, the concept of intuitionistic fuzzy set proposed by Krassimir [29] considers three aspects of information: membership, non-membership, and hesitancy. For language evaluations that cannot be represented numerically, they can be transformed into intuitionistic fuzzy numbers.

The intuitionistic fuzzy set correlation calculations are as follows:

A={<x,m(x),n(x)>|x∈Z}

where m(x), n(x) are the affiliation and non-affiliation functions of A, respectively. If x all belong to Z and

0≤m(x)+n(x)≤1,0≤m(x)≤1,0≤n(x)≤1,m(x)+n(x)+π(x)=1,0≤π(x)≤1, where π(x) is the hesitancy degree of A, then we call (m(x),n(x)) a normalized intuitionistic fuzzy number. For more on intuitionistic fuzzy number, we can refer to Sivaraman G et al. [30] and Hayat K et al. [31], Hossein G et al. [32].

If there exist two intuitionistic fuzzy numbers $A_{1}=\left(m_{1},n_{1}\right)$ and $A_{2}=\left(m_{2},n_{2}\right)$, then the algorithms are as follows:

$$A_{1}⊕A_{2}=\left(m_{1}+m_{2}-m_{1}m_{2},n_{1}n_{2}\right)$$

$$A_{1}⊗A_{2}=\left(m_{1}+m_{2},n_{1}+n_{2}-n_{1}n_{2}\right)$$

$$\lambda A_{1}=\left(1-{\left(1-m_{1}\right)}^{\lambda },n_{1}^{\lambda }\right)$$

$${A_{1}}^{\lambda }=\left(m_{1}^{\lambda },1-{\left(1-n_{1}\right)}^{\lambda }\right)$$

And the Hamming distance is: $D\left(A_{1},A_{2}\right)=0.5\left(\left|m_{1}-m_{2}\right|+\left|n_{1}-n_{2}\right|+\left|\pi_{1}-\pi_{2}\right|\right)$, where π is the hesitation.

There are some differences between intuitionistic fuzzy set theory and the other fuzzy set theories. Intuitionistic fuzzy set theory increases the hesitancy degree and can be described from three perspectives of support, opposition and abstention at the same time, which makes the result more reasonable and reliable. Additionally, the integration of intuitionistic fuzzy set theory can enhance the model's correctness and dependability, more effectively enable decision-making and problem-solving, and more accurately capture the fuzziness and uncertainty in real-world scenarios.

(2) QOS data processing

Real numbers, interval numbers, and evaluation linguistic variables are examples of QOS data types in this paper that need to be converted into intuitionistic fuzzy numbers in various formats. Table 1 is used to convert the evaluation linguistic variables into intuitionistic fuzzy numbers, and the following methods are used to convert real numbers and interval numbers.

①. Obtain the initial decision matrix $Z={\left(z_{ij}\right)}_{m\times n}$.
②. The initial decision matrix Z is normalized, and all of them are transformed into positive decision matrix $Z_{1}$.
③ Based on the positive decision matrix $Z_{1}$, the quasi-satisfactory value $A^{\prime }$, the quasi-unsatisfactory value B,and the quasi-uncertain value C of $z_{ij}^{\prime }$ are calculated according to the following formulas. For two numeric data $a_{1}$， $b_{1}$, $d=\left|a_{1}-b_{1}\right|$ is the distance between them; for two interval data $\left[a_{1},a_{2}\right]$， $\left[b_{1},b_{2}\right]$, $d=\frac{1}{2}\left(\left|a_{1}-b_{1}\right|+\left|a_{2}-b_{2}\right|\right)$ is the distance between them.

$$A^{\prime }=\left\{ \begin{array}{c} z_{ij}^{，},d\left(z_{ij}^{，},\max\;z_{ij}^{，}\right)＜d\left(z_{ij}^{，},\min\;z_{ij}^{，}\right);\\ midz_{ij}^{，},d\left(z_{ij}^{，},\max\;z_{ij}^{，}\right)\geq d\left(z_{ij}^{，},\min\;z_{ij}^{，}\right); \end{array}\right.$$

$$B=\left\{ \begin{array}{c} z_{ij}^{，},d\left(z_{ij}^{，},\max\;z_{ij}^{，}\right)＞d\left(z_{ij}^{，},\min\;z_{ij}^{，}\right);\\ midz_{ij}^{，},d\left(z_{ij}^{，},\max\;z_{ij}^{，}\right)\leq d\left(z_{ij}^{，},\min\;z_{ij}^{，}\right); \end{array}\right.$$

$$C=\left(A^{\prime }+B\right)/2$$

$$\max\;z_{ij}^{，}=\left\{ \begin{array}{c} \max\;z_{ij}^{，}，\text{the}\;\text{index}\;\text{is}\;\text{numeric}\;\text{data};\\ \left(\max\;z_{ij}^{，}，\max\;z_{ij}^{，}\right)，\text{the}\;\text{metric}\;\text{is}\;\text{interval}\;\text{data}; \end{array}\right.$$

$$\min\;z_{ij}^{，}=\left\{ \begin{array}{c} \min\;z_{ij}^{，}，\text{the}\;\text{index}\;\text{is}\;\text{numeric}\;\text{data}\\ \left(\min\;z_{ij}^{，}，\min\;z_{ij}^{，}\right)，\text{the}\;\text{metric}\;\text{is}\;\text{interval}\;\text{data}; \end{array}\right.$$

$$midz_{ij}^{，}=\left(\max\;z_{ij}^{，}+\min\;z_{ij}^{，}\right)/2$$

④. Calculate The quasi-membership degree D, quasi-non-membership degree E and quasi-hesitation degree F based on the following formulas.

$$D=\frac{d\left(A,midz_{ij}^{，}\right)}{d\left(A,midz_{ij}^{，}\right)+d\left(A,\max\;z_{ij}^{，}\right)}$$

$$E=\frac{d\left(B,midz_{ij}^{，}\right)}{d\left(B,midz_{ij}^{，}\right)+d\left(B,\min\;z_{ij}^{，}\right)}$$

$$F=\left\{ \begin{array}{c} \frac{d\left(B,C\right)}{d\left(B,C\right)+d\left(C,midz_{ij}^{，}\right)},d\left(C,\max\;z_{ij}^{，}\right)＞d\left(C,\min\;z_{ij}^{，}\right)\\ \frac{d\left(A,C\right)}{d\left(A,C\right)+d\left(C,midz_{ij}^{，}\right),}d\left(C,\max\;z_{ij}^{，}\right)＜d\left(C,\min\;z_{ij}^{，}\right)\\ 1，d\left(C,\max\;z_{ij}^{，}\right)=d\left(C,\min\;z_{ij}^{，}\right) \end{array}\right.$$

⑤. Calculate the membership degree, non-membership degree and hesitancy degree based on the formula to form the intuitionistic fuzzy matrix $B={\left(b_{ij}\right)}_{m\times n}={\left(m_{ij},n_{ij}\right)}_{m\times n}$.

$$m_{ij}=\left\{ \begin{array}{c} \frac{D}{D+1/2F},d\left(z_{ij},\max\;z_{ij}^{，}\right)＜d\left(z_{ij},\min\;z_{ij}^{，}\right);\\ 0,d\left(z_{ij},\max\;z_{ij}^{，}\right)\geq d\left(z_{ij},\min\;z_{ij}^{，}\right); \end{array}\right.$$

$$n_{ij}=\left\{ \begin{array}{c} \frac{E}{E+1/2F},d\left(z_{ij},\max\;z_{ij}^{，}\right)＜d\left(z_{ij},\min\;z_{ij}^{，}\right);\\ 0,d\left(z_{ij},\max\;z_{ij}^{，}\right)\geq d\left(z_{ij},\min\;z_{ij}^{，}\right); \end{array}\right.$$

$$\pi_{ij}=1-m_{ij}-n_{ij}$$

**Table 1:** Correspondence of evaluation linguistic variables - intuitionistic fuzzy number.

evaluation linguistic variables	intuitionistic fuzzy number
VG	[0.95,0.02]
G	[0.75,0.15]
M	[0.45,0.45]
P	[0.15,0.75]
VP	[0.02.0.95]

### 3.2 QOS index integration empowerment

This paper proposes an integrated assignment method based on the principle of maximum entropy. Firstly, the subjective and objective weights of QOS evaluation indexes are assigned by the G1-method and the variable precision rough set theory; then, based on the combination coefficients of the maximum entropy principle, the subjective and objective weights are integrated to form the final combination weights.

(1) G1-method to determine subjective weights.

The G1-method assigns weights to response indicators by subjective ranking and pairwise comparison. The steps are as follows.

①. Determine the subjective weights of the QOS evaluation metrics in this paper and rank them in ascending order: $s_{1}<s_{2}<s_{3}...<s_{m}$.
②. According to the neighboring indicators given in Table 1, the relative importance is assigned. It is shown as Table 2:

**Table 2:** $g_{k}$ Meaning.

$g_{k}$	meaning
1.0	Both are equally important
0.7	The latter is slightly more important than the former
0.5	The latter is moderately more important than the former
0.2	The latter is vastly more important than the former
③. Calculate the weight coefficient $\omega_{k}$, that is:

(1) $$\omega_{k+1}=\frac{1}{1+\sum_{k=1}^{m}\prod_{i=k}^{m}g_{k+1}}$$

(2) $$\omega_{k}=g_{k+1}\omega_{k+1}\;\left(k=1,2,3...m\right)$$

According to the formula, the weight $\omega_{10}$ of the index $s_{10}$ can be obtained, and then by substituting the above formula, we can obtain: $\omega_{9}$， $\omega_{8}$， $\omega_{7}$， ….

(2) Variable precision rough set theory to determine the objective weight.

In this paper, variable precision rough set theory is used to objectively weight the QOS evaluation indexes.

The decision table of shared manufacturing service evaluation information is as follows:

C=(U,S∪W)

where U is a non-empty subset of feasible services; S is the set of indicator attributes; W is the set of decision attributes.

Suppose that the shared manufacturing feasible service set U is the equivalent class which is divided by index attribute S, and the shared manufacturing feasible service set U is the equivalent class which is divided by decision attribute W. If $X_{h}\subseteq Y_{h}$ is present, the relative misclassification rate for:

$$e\left(X_{h},Y_{h}\right)=1-\frac{\left|X_{h}\cap Y_{h}\right|}{\left|X_{h}\right|}$$

$$X_{h}=\frac{U}{S}=\left\{X_{1},X_{2},X_{3}...X_{\left|\frac{U}{S}\right|}\right\}$$

$$Y_{h}=\frac{U}{W}=\left\{X_{1},X_{2},X_{3}...X_{\left|\frac{U}{W}\right|}\right\}$$

where $\left|X_{h}\right|$ is the number of equivalence classes $X_{h}$ manufacturing service combinations.

Let α ∈[0.0.5], then the $Y_{h}$ of the lower approximation for S is:

$$pos_{xh}^{\alpha }\left(Y_{h}\right)=\cup \left(Y_{h}=\frac{u}{w}|e\left(X_{h},Y_{h}\right)\leq \alpha \right)$$

The amount of information sharing the attributes of a manufacturing service index indicates the ability of the index to classify a set of composite services. The greater the amount of information, the stronger the ability of classification, the information content (β)of any service indicator ($S_{m}$) is:

$$\beta \left(S_{m}\right)=\frac{1}{{\left|U\right|}^{2}}\sum_{h=1}^{\frac{U}{S}}X_{h}^{2}\;S_{m}\in S$$

The degree of dependence of shared manufacturing service index attribute indicates the degree of dependence of the index attribute on the classification of decision attributes, The degree of dependency (γ)of any of the service indicators ($S_{m}$) is:

$$\gamma \left(S_{m}\right)=\frac{\sum_{h=1}^{\frac{U}{W}}pos_{xh}^{\alpha }\left(Y_{h}\right)}{U}$$

In summary, the objective weight of the service indicator attributes is:

(3) $$\omega_{j}=\frac{\beta \left(S_{m}\right)\times \gamma \left(S_{m}\right)}{\sum_{m=1}^{m}\beta \left(S_{m}\right)\times \gamma \left(S_{m}\right)}$$

(3) Determining combination weight based on maximum entropy.

This paper adopts the additive assignment method based on the principle of maximizing entropy. Under the premise of known service indicator information, the reasonable distribution of probability can be obtained by maximizing entropy; generalized distance sum is the weighted distance sum of the indicator parameter values and ideal values of the shared manufacturing service combination scheme, so the combination coefficient of weights $\delta_{\tau }$ is obtained by using maximizing entropy and generalized distance sum.

Construct a composite coefficient model:

(4) $$\max\;g\left(\delta \right)=-\sum_{\tau =1}^{2}\delta_{\tau }\;\ln\;\delta_{\tau }$$

$$\left\{ \begin{array}{c} \sum_{\varepsilon =1}^{Z}\sum_{\theta =1}^{m}\sum_{\tau =1}^{2}\delta_{\tau }\omega_{\theta }^{\tau }\left(1-\gamma_{\varepsilon \theta }\right)=D_{s}\\ \sum_{\tau =1}^{2}\delta_{\tau }=1 \end{array}\right.$$

$\varphi_{1}$ is the Lagrange multiplier. Constructing the Lagrange function and solving it by pairwise programming, the pairwise programming is as follows:

(5) $$\min\;f\left(\varphi_{1}\right)=\ln\sum_{\tau =1}^{2}\exp\left[\varphi_{1}\sum_{\varepsilon =1}^{Z}\sum_{\theta =1}^{m}\omega_{\theta }^{\tau }\left(1-r_{\varepsilon \theta }\right)\right]-\varphi_{1}D_{s}$$

Substitute the obtained $\varphi_{1}$ into the following equation to find the combination coefficient $\delta_{\tau }$:

(6) $$\delta_{\tau }=\frac{\exp\left[\varphi_{1}\sum_{\varepsilon =1}^{Z}\sum_{\theta =1}^{m}\omega_{\theta }^{\tau }\left(1-r_{\varepsilon \theta }\right)\right]}{\sum_{\tau =1}^{2}\exp\left[\varphi_{1}\sum_{\varepsilon =1}^{Z}\sum_{\theta =1}^{m}\omega_{\theta }^{\tau }\left(1-r_{\varepsilon \theta }\right)\right]}$$

Therefore, the combined weight of the service indicators is:

(7) $$\omega =\delta_{\tau }\omega_{k}+\delta_{\tau }\omega_{j}$$

### 3.3 Constructing a service recommendation model

A mathematical model for the multi-objective optimization problem of shared manufacturing services is constructed with service availability, service reliability, security, credibility, information sharing capability, quality, green manufacturing capability, best flexibility, least cost and shortest time as the objectives, and dual constraints of supply and demand including payment speed of the demand users and credibility index as the constraints.

(8) MaxF=(t,c,sav,sre,ssa,scre,insc,qua,sfle,grca)

$$\left\{ \begin{array}{c} s=c,t,sav,sre,ssa,scre,insc,qua,sfle,grca\\ q_{ns}\leq q_{s}^{\max},s=c,t\\ q_{ns}\geq q_{s}^{\min},s=sav,sre,ssa,scre,insc,qua,sfle,grca\\ dps\geq dps_{n}^{\min}\\ dcy\geq dcy_{n}^{\min}\\ n=1,2,3... \end{array}\right.$$

Where F is the total objective optimization function. The Pareto optimal solution set of the service evaluation problem is solved by building a multi-objective optimization model.

### 3.4 Evaluation of shared manufacturing services using the VIKOR methodology

The evaluation of shared manufacturing services is divided into two parts: the multi-objective optimization problem to solve the Pareto-optimal solution set and the comprehensive evaluation value to calculate the ranking. Based on the optimal solution set derived above, for the candidate services in the optimal solution set, based on intuitionistic fuzzy matrix $B={\left(b_{ij}\right)}_{m\times n}={\left(m_{ij},n_{ij}\right)}_{m\times n}$, this paper adopts VIKOR evaluation method, VIKOR is a compromise evaluation method, and it is a compromise solution proposed to rank a group of alternatives relative to the conflict criterion, in which the maximum combined benefit values and the minimum regret values are calculated, and VIKOR will make the decision more reasonable and reliable. Therefore, this paper chooses VIKOR Analysis Method for service evaluation. The steps are as follows:

Step 1. Based on the following formula, the maximum combined benefit values and the minimum regret values of the shared manufacturing services are solved.

(9) $$S_{i}=\sum_{j=1}^{n}\omega \frac{d\left(b_{j}^{+},b_{ij}\right)}{d\left(b_{j}^{+},b_{j}^{-}\right)}$$

(10) $$R_{i}=\max\;\omega \frac{d\left(b_{j}^{+},b_{ij}\right)}{d\left(b_{j}^{+},b_{j}^{-}\right)}$$

where $b_{j}^{+}$ denotes the best value of the indicator S and $b_{j}^{-}$ denotes the worst value of the indicator S.

Step 2. Calculate the composite index $Q_{i}$.

(11) $$Q_{i}=v\frac{S_{i}-S_{i}^{-}}{S_{i}^{+}-S_{i}^{-}}+\left(1-v\right)\frac{R_{i}-R_{i}^{-}}{R_{i}^{+}-R_{i}^{-}}\;i=1,2,3...$$

Where v >0.5 indicates that $S_{i}$ accounts for a larger proportion, v <0.5 indicates that $R_{i}$ accounts for a larger proportion, therefore, v = 0.5 is in this paper.

Step 3. The evaluation ranking of the shared manufacturing service providers is obtained by ranking the $Q_{i}$ values in descending order.
Step 4. Simulate the evaluation method and verify the feasibility of VIKOR.

## 4 Numerical analysis

To verify the validity of the research methodology studied in this paper, a domestic industrial Internet intelligent manufacturing platform is taken as a numerical example, which is a unified operation platform for mass customization of products by users such as equipment manufacturers and manufacturing enterprises. Taking a machine tool manufacturer in the platform as an example, the customer submits the manufacturing request to the platform, and the platform completes the search in the platform resource base according to the service request. It supposes that the platform processing request center matches 30 suppliers to satisfy the request, in which the customer is the demander of shared manufacturing service and the supplier is the provider of shared manufacturing service; then the multi-objective optimization model based on supply and demand constraints is solved, and 12 Pareto efficiency solution sets are output. According to the demands of both supply and demand, the platform selects 12 shared manufacturing services from the service set($SS_{1}$， $SS_{2}$， $SS_{3}$， $SS_{4}$， $SS_{5}$， $SS_{6}$， $SS_{7}$， $SS_{8}$， $SS_{9}$， $SS_{10}$， $SS_{11}$， $SS_{12}$), the initial decision matrix is constructed by combining the online and offline QOS indicators, as shown in Table 3.

**Table 3:** Initial decision matrix.

Shared Manufacturing Services	$S_{1}$	$S_{2}$	$S_{3}$	$S_{4}$	$S_{5}$	$S_{6}$	$S_{7}$	$S_{8}$	$S_{9}$	$S_{10}$
${SS}_{1}$	0.88	0.96	G	M	VG	68	[79,85]	0.96	G	M
${SS}_{2}$	0.86	0.97	G	G	VG	60	[75,78]	0.93	G	G
${SS}_{3}$	0.98	0.92	G	VG	G	65	[76,82]	0.92	G	M
${SS}_{4}$	0.89	0.96	M	M	G	58	[75,83]	0.92	G	M
${SS}_{5}$	0.94	0.92	VG	G	G	69	[79,87]	0.97	G	G
${SS}_{6}$	0.98	0.93	VG	G	G	62	[75,83]	0.94	VG	M
${SS}_{7}$	0.95	0.95	G	VG	VG	61	[79,82]	0.96	VG	G
${SS}_{8}$	0.93	0.91	M	G	M	70	[75,78]	0.93	G	M
${SS}_{9}$	0.95	0.95	VG	VG	VG	63	[76,80]	0.99	G	VG
${SS}_{10}$	0.95	0.99	G	VG	VG	61	[75,85]	0.95	G	G
${SS}_{11}$	0.94	0.97	VG	G	G	62	[83,85]	0.99	G	G
${SS}_{12}$	0.91	0.95	G	VG	G	60	[85,87]	0.95	VG	M

**Table 4:** Decision matrix for positivity.

Shared Manufacturing Services	$S_{1}$	$S_{2}$	$S_{3}$	$S_{4}$	$S_{5}$	$S_{6}$	$S_{7}$	$S_{8}$	$S_{9}$	$S_{10}$
${SS}_{1}$	0.88	0.96	G	M	VG	68	[2,8]	0.96	G	M
${SS}_{2}$	0.86	0.97	G	G	VG	60	[9,12]	0.93	G	G
${SS}_{3}$	0.98	0.92	G	VG	G	65	[5,11]	0.92	G	M
${SS}_{4}$	0.89	0.96	M	M	G	58	[4,12]	0.92	G	M
${SS}_{5}$	0.94	0.92	VG	G	G	69	[0, 8]	0.97	G	G
${SS}_{6}$	0.98	0.93	VG	G	G	62	[4,12]	0.94	VG	M
${SS}_{7}$	0.95	0.95	G	VG	VG	61	[5,8]	0.96	VG	G
${SS}_{8}$	0.93	0.91	M	G	M	70	[9,12]	0.93	G	M
${SS}_{9}$	0.95	0.95	VG	VG	VG	63	[7,11]	0.99	G	VG
${SS}_{10}$	0.95	0.99	G	VG	VG	61	[2,12]	0.95	G	G
${SS}_{11}$	0.94	0.97	VG	G	G	62	[2,4]	0.99	G	G
${SS}_{12}$	0.91	0.95	G	VG	G	60	[0, 2]	0.95	VG	M

### 4.1 Example solving

(1) The initialization matrix is normalized to obtain the positivity decision matrix as shown in Table 4.

(2) According to the above formula, the intuitionistic fuzzy matrix is obtained as shown in Table 5.

(3) Calculation of weights

Based on the intuitionistic fuzzy matrix derived above, the subjective weights are first determined using the G1-method, and then the objective weights are determined using the variable precision rough set theory, and finally the combined weight values of each attribute of QOS are derived. The subjective weight values $\omega_{k}$ for each attribute based on the above equation are: {0.085, 0.104, 0.062, 0.055, 0.049, 0.165, 0.132, 0.236, 0.044, 0.068}; the objective weight values $\omega_{j}$ are: {0.091, 0.115, 0.084, 0.071, 0.057, 0.120, 0.147, 0.182, 0.047, 0.086}; Using MATLAB to simulate the principle of maximum entropy combination weighting model, the generalized distance sum of 3.743, the combination coefficients of 0.429 and 0.571 are selected from the results, and substituting them into the formulas, the combined weights of the 10 indexes are: {0.09,0.11,0.075,0.064,0.058,0.129,0.14,0.21,0.046,0.078}.

(4) According to the intuitionistic fuzzy matrix and the weight of each index, calculating the combination benefit and minimum regret value is obtained as shown in Table 6.

(5) The composite metrics are calculated, and the shared manufacturing services are ranked as follows:

**Table 5:** Intuitionistic fuzzy matrix.

Services	S_1_	S_2_	S_3_	S_4_	S_5_	S_6_	S_7_	S_8_	S_9_	S_10_
${SS}_{1}$	[0.0]	[0.5.0]	[0.75.0.15]	[0.45,0.45]	[0.95.0.02]	[0.0.727]	[0.0.727]	[0.364.0]	[0.75.0.15]	[0.45,0.45]
${SS}_{2}$	[0.0]	[0.667.0]	[0.75.0.15]	[0.75.0.15]	[0.95.0.02]	[0.727.0]	[0.727.0]	[0.0]	[0.75.0.15]	[0.75.0.15]
${SS}_{3}$	[0.8.0]	[0.0]	[0.75.0.15]	[0.95.0.02]	[0.75.0.15]	[0.0.4]	[0.0.4]	[0.0]	[0.75.0.15]	[0.45,0.45]
${SS}_{4}$	[0.0]	[0.5.0]	[0.45,0.45]	[0.45,0.45]	[0.75.0.15]	[0.8.0]	[0.8.0]	[0.0]	[0.75.0.15]	[0.45,0.45]
${SS}_{5}$	[0.541.0]	[0.0]	[0.95.0.02]	[0.75.0.15]	[0.75.0.15]	[0.0.769]	[0.0.640]	[0.632.0]	[0.75.0.15]	[0.75.0.15]
${SS}_{6}$	[0.8.0]	[0.0]	[0.95.0.02]	[0.75.0.15]	[0.75.0.15]	[0.571.0]	[0.372.0]	[0.0]	[0.95.0.02]	[0.45,0.45]
${SS}_{7}$	[0.667.0]	[0.0]	[0.75.0.15]	[0.95.0.02]	[0.95.0.02]	[0.667.0]	[0.372.0]	[0.364.0]	[0.95.0.02]	[0.75.0.15]
${SS}_{8}$	[0.4.0]	[0.0]	[0.45,0.45]	[0.75.0.15]	[0.45,0.45]	[0.0.8]	[0.602.0]	[0.0]	[0.75.0.15]	[0.45,0.45]
${SS}_{9}$	[0.667.0]	[0.0]	[0.95.0.02]	[0.95.0.02]	[0.95.0.02]	[0.4.0]	[0.508.0]	[0.8.0]	[0.75.0.15]	[0.95.0.02]
${SS}_{10}$	[0.667.0]	[0.8.0]	[0.75.0.15]	[0.95.0.02]	[0.95.0.02]	[0.667.0]	[0.372.0]	[0.0]	[0.75.0.15]	[0.75.0.15]
${SS}_{11}$	[0.571.0]	[0.667.0]	[0.95.0.02]	[0.75.0.15]	[0.75.0.15]	[0.571.0]	[0.0.674]	[0.8.0]	[0.75.0.15]	[0.75.0.15]
${SS}_{12}$	[0.0]	[0.0]	[0.75.0.15]	[0.95.0.02]	[0.75.0.15]	[0.727.0]	[0.0.671]	[0.0]	[0.95.0.02]	[0.45,0.45]

**Table 6:** Combined benefits and minimum regret value.

candidate shared manufacturing services	combined benefit values	minimum regret values
${SS}_{1}$	0.7248	0.1400
${SS}_{2}$	0.4552	0.2100
${SS}_{3}$	0.6993	0.2100
${SS}_{4}$	0.6262	0.2100
${SS}_{5}$	0.5601	0.1320
${SS}_{6}$	0.5004	0.2100
${SS}_{7}$	0.3450	0.1145
${SS}_{8}$	0.7932	0.2000
${SS}_{9}$	0.2314	0.1100
${SS}_{10}$	0.3766	0.2100
${SS}_{11}$	0.3160	0.1351
${SS}_{12}$	0.6773	0.2100

The composite metrics $Q_{i}$ of the candidate shared manufacturing services are calculated according to the formula as: {0.5891, 0.6992, 0.9164, 0.8514, 0.4025, 0.7394, 0.1011, 0.9500, 0, 0.6292, 0.2008, 0.8968}.

The final scores of the candidate services (1-$Q_{i}$)are obtained as shown in Table 7.

**Table 7:** Final ranking of services.

candidate shared manufacturing services	1-$Q_{i}$	Sort
${SS}_{1}$	0.4109	5
${SS}_{2}$	0.3008	7
${SS}_{3}$	0.0836	11
${SS}_{4}$	0.1486	9
${SS}_{5}$	0.5975	4
${SS}_{6}$	0.2606	8
${SS}_{7}$	0.8989	2
${SS}_{8}$	0.0500	12
${SS}_{9}$	1.0000	1
${SS}_{10}$	0.3708	6
${SS}_{11}$	0.7992	3
${SS}_{12}$	0.1036	10

From the above Table 7, the optimal shared manufacturing service provider obtained by using the VIKOR method is $SS_{9}$, the least selected as shared manufacturing service provider is $SS_{8}$. Analyzing the attributes of the indicators in this paper, the weights of cost, quality, and green manufacturing capability are higher, and relative to the second-best service provider $SS_{3}$, the cost, quality, and green manufacturing capability of optimal service provider $SS_{9}$ are far better than the second-best service provider's, with lower cost, higher quality, and more green manufacturing capability (more environmental protection in the manufacturing process). Cost, turnaround time, and quality are still crucial benchmarks for determining manufacturing capacity in the modern industrial sector. When selecting service providers, many superior manufacturers have raised their standards considering their green manufacturing capabilities. The key is a competent service provider who is both affordable and of great quality, but it's also important to consider their capacity for green production.

Therefore, this paper adopts the method to select the appropriate shared manufacturing service provider for the user.

### 4.2 Sensitivity analysis

In actual decision-making, different decision-making attitudes will take different compromise coefficients v.With the change of v, the scheme will be affected. The choice mechanism coefficient v=0.5 was used to generate the current outcome, indicating that the decision maker's preference value for the largest combination benefit and the lowest regret value is the same. While the sensitivity analysis aims to the decision-making mechanism coefficient is to perturb the decision-making mechanism coefficient, so that it is in the [0, 1] interval changes in the value of the program, consider the decision maker's preference situation for all S-value and R-value, to better reflect the stability of decision-making results and the reliability of program selection.

**Fig. 4:**
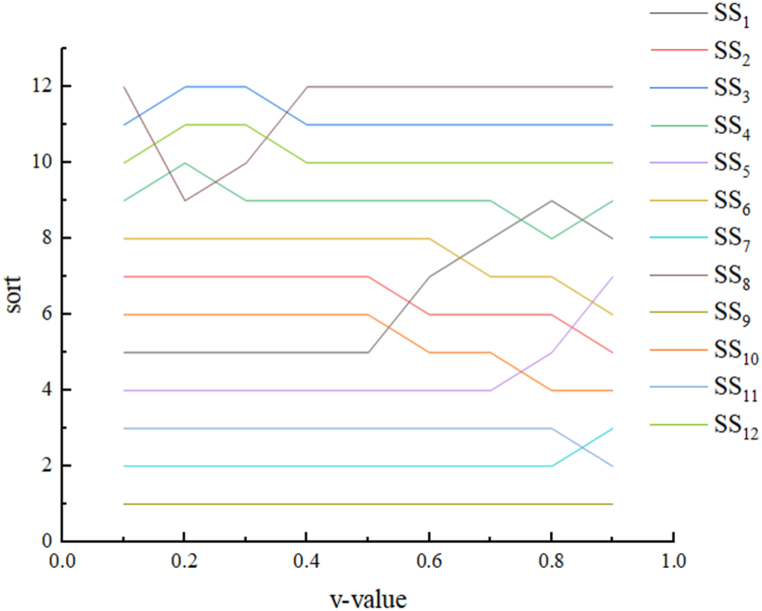
Experimental results for different values of v.

This paper takes the ratio of the utility interest and the minimum regret value obtained from the case study (the value of v is taken in the formula) to change on the experimental results to carry out a sensitivity analysis. With a step size of 0.1 and 9 fetches in [0, 1], the stability of the previous decision-making scheme can be judged by the ranking result of Q after disturbance. Taking v as {0.1, 0.2, 0.3 … 0.9} for the calculation, respectively, the sorting results and the experimental results are shown in Fig. 4 it can be seen in Fig. 4.

We can see that the Q-value of $SS_{9}$ is always the smallest, and $SS_{9}$ has sufficient stability when influenced by the decision maker's preference for the largest combination benefit and the lowest regret value, and at the same time, when the value of the v is changed, the top 4 ranked shared manufacturing services do not undergo any large changes, and all the changed shared manufacturing services changed in relatively small intervals. Therefore, we can conclude that the optimal shared manufacturing services derived from the VIKOR method used in this paper do not change with the adjustment of the coefficients of the decision-making mechanism and have good reliability.

### 4.3 Comparative analysis

To verify the validity and superiority of the combination assignment-VIKOR method, the method in the paper is compared with other commonly used decision-making methods such as TOPSIS method and grey correlation method.

(1) TOPSIS method

Firstly, according to the optimal value of each index attribute to determine its positive ideal solution and the worst value to determine its negative ideal solution, and then use the Euclidean distance to determine the gap between the candidate shared manufacturing services and the positive and negative ideal solutions, and finally calculate the relative closeness between each candidate service and the positive and negative ideal solutions. The calculation results are shown in Table 8 below.

(2) Grey correlation method

**Table 8:** Comparison of ranking results obtained using different methods.

Evaluation methods	${SS}_{1}$	${SS}_{2}$	${SS}_{3}$	${SS}_{4}$	${SS}_{5}$	${SS}_{6}$	${SS}_{7}$	${SS}_{8}$	${SS}_{9}$	${SS}_{10}$	${SS}_{11}$	${SS}_{12}$
assignment-VIKOR method	0.4109	0.3008	0.0836	0.1486	0.5975	0.2606	0.8989	0.0500	1.0000	0.3708	0.7992	0.1036
Sort	5	7	11	9	4	8	2	12	1	6	3	10
${SS}_{9}>{SS}_{7}>{SS}_{11}>{SS}_{5}>{SS}_{1}>{SS}_{10}{>SS}_{2}>{SS}_{6}>{SS}_{4}{>SS}_{12}>{SS}_{3}>{SS}_{8}$												
Topsis method	0.3414	0.5447	0.4171	0.4640	0.4347	0.5119	0.5833	0.3322	0.6364	0.6254	0.6344	0.3782
Sort	11	5	9	7	8	6	4	12	1	3	2	10
${SS}_{9}>{SS}_{11}>{SS}_{10}>{SS}_{7}>{SS}_{2}>{SS}_{6}{>SS}_{4}>{SS}_{5}>{SS}_{3}{>SS}_{12}>{SS}_{1}>{SS}_{8}$												
Grey correlation degree method	0.4478	0.6066	0.4750	0.5526	0.5354	0.5615	0.6231	0.4092	0.7485	0.6352	0.6840	0.5053
Sort	11	5	10	7	8	6	4	12	1	3	2	9
${SS}_{9}>{SS}_{11}>{SS}_{10}>{SS}_{7}>{SS}_{2}>{SS}_{6}{>SS}_{4}>{SS}_{5}>{SS}_{12}{>SS}_{3}>{SS}_{1}>{SS}_{8}$												

First find out the positive optimal object $\left\{X_{\max}\right\}$ in the intuitionistic fuzzy set, then calculate the association coefficient between each array $\left\{X_{i}\right\}$ and the positive optimal object $\left\{X_{\max}\right\}$ according to the following formula, and calculate the correlation degree λ, which is optimal for the ranking of candidate services. The calculation results are shown in Table 8.

From the above table, the optimal service is $SS_{9}$, and the worst service is $SS_{8}$. As can be seen from the above calculations, the optimal service derived from the method proposed in this paper is consistent with the optimal service derived from common decision-making methods, indicating that the method is effective in evaluating the selection of service providers. Of course, it is unappreciated to say that one method is better or powerful than the other one, because every method has different unique features developed under different theories. However, given the volatility of the index weights and the complexity of the model and so on, the VIKOR evaluation method based on intuitionistic fuzzy is more accurate and more suitable than TOPSIS and grey relational degree method.

On the volatility of the index weights: in the actual situation, the index weight value will change under uncertain factors, when the index weight changes to {0.1,0.05,0.042,0.05,0.035,0.18,0.22, 0.15,0.128, 0.045}, increasing the cost-time quality and the proportion of green manufacturing capacity, the results in a ranking of services are shown in Table 9.

**Table 9:** Comparison of the results after changing the weights.

Evaluation methods	Sort
assignment-VIKOR method	${SS}_{9}>{SS}_{11}>{SS}_{7}>{SS}_{5}>{SS}_{1}>{SS}_{2}{>SS}_{10}>{SS}_{6}>{SS}_{3}{>SS}_{12}>{SS}_{4}>{SS}_{8}$
Topsis method	${SS}_{7}>{SS}_{11}>{SS}_{9}>{SS}_{5}>{SS}_{1}>{SS}_{10}{>SS}_{4}>{SS}_{6}>{SS}_{2}{>SS}_{12}>{SS}_{3}>{SS}_{8}$
Grey correlation degree method	${SS}_{5}>{SS}_{9}>{SS}_{11}>{SS}_{7}>{SS}_{4}>{SS}_{10}{>SS}_{2}>{SS}_{1}>{SS}_{12}{>SS}_{8}>{SS}_{3}>{SS}_{6}$

As can be seen from Table 9 above, the ranking results obtained by the VIKOR method do not change much in the case of changing the weights of the indicators, while the ranking results obtained by the TOPSIS method and the grey correlation method show a large change, which is due to the fact that the TOPSIS method and the Grey correlation method are more sensitive to errors or uncertainties, which will have a large impact on the evaluation results, and are not very suitable for the analysis of multi-indicator decision-making.

**Fig. 5:**
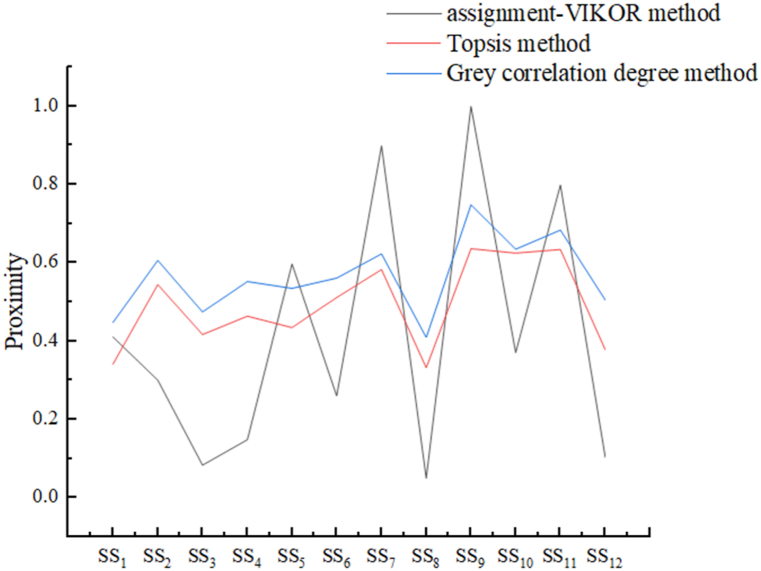
Comparison of closeness under different methods.

To compare the advantages and disadvantages of the methods more intuitively, the attribute proximity comparison of the three methods for shared manufacturing services is plotted, as shown in Fig. 5.

As can be seen in Fig. 5 above, the approach used in this paper is superior to the other two approaches, and the gap between the evaluated values obtained by the VIKOR method is larger, so the optimal solution obtained is closer to the ideal solution.

On the complexity of the model: 1) compared with the grey correlation method, the correlation value is obtained by inviting several experts to score when carrying out the grey correlation method, which results in a higher degree of subjectivity and less variability in the decision-making results; and when there are a very large number of decision-making options, models are getting more and more complex, the method is not conducive to the decision-maker to make a reasonable judgment; 2) compared with the TOPSIS method, the difference between the evaluated values is small, resulting in not necessarily the best program, while taking the method in this paper, based on the final comparison of the comprehensive utility values of the programs to rank the advantages and disadvantages of the programs and the larger gap between the evaluated values of different programs, makes that the optimal program is closer to the ideal program. Especially in the case of many programs, models are getting more complex, the advantage of the VIKOR method is more obvious.

## 5 Conclusions

It is a crucial task to evaluate many shared manufacturing services with similar function attributes and select the best service to form the best shared manufacturing service composition. In this paper, the QOS evaluation index system under shared manufacturing service is constructed, which is based on the evaluation of the candidate service providers and the selection of the best service to meet the requirements of the user, a comprehensive evaluation of the candidate services is carried out and the main conclusions are as follows:(1) As can be seen from the comparative analysis, considering the fluctuation of the index weights and the complexity of the model, etc., the intuitionistic fuzzy-based VIKOR evaluation method proposed in this paper tends to produce more accurate and appropriate results than TOPSIS and grey correlation methods, and the optimal solution obtained is closer to the ideal solution, which is a more obvious advantage. (2) From the sensitivity analysis, it can be seen that the optimal shared manufacturing service derived from the intuitionistic fuzzy-based VIKOR evaluation method used in this paper does not change with the adjustment of the decision-making mechanism coefficients and has good reliability.(3) Construct the QOS evaluation system from both online and offline aspects, which is divided into 2 first-level indicators and 10 second-level indicators, and analyze the examples to conclude that the cost, quality, service reliability, time and green manufacturing capability are the key influencing factors, which are also the indicator parameters to be focused on in the future work.

The significance of this paper is mainly as follows: the openness of the sharing platform, the wide range of service providers, and the complexity of users' demands make users' matching and selection of services from a large number of manufacturing resources become the key to the efficient development of shared manufacturing, and the proposal of a new evaluation method provides an additional choice for demand users to select a satisfactory service provider, provides users with dynamic, on-demand supply and high-quality services, improve the efficiency of enterprises, and reduce costs and indirectly realize green and low-carbon manufacturing; an efficient supply and demand matching mechanism makes the shared manufacturing business with the change of service scale can be flexibly and quickly composed of various types of service resources to meet the user's needs, and to achieve the purpose of agile manufacturing.

The limitations and prospects of this paper is mainly as follows: Firstly, this paper only considers a single shared manufacturing service to meet user needs, but in practice, with the complexity of the production task, a single service can no longer fully meet the users' needs. The next step will be an in-depth study of "many-to-many" matching between shared manufacturing services and tasks. Secondly, we need to find new ways to adapt to complex situations, such as GGIFSSs proposed by Hayat K et al. [33] and IVq-ROFSSs proposed by Yang et al. [34]. Thirdly, another evaluation algorithm is developed to address the evaluation problem of shared manufacturing services.

## Funding

This work was supported by the Research Project on Economic and Social Development of Liaoning Province under Grant 2024lslybkt-058, Liaoning Graduate Education and Teaching Reform Research Project under Grant LNYJG2022062. The authors wish to acknowledge the contribution of Liaoning Key Lab of Equipment Manufacturing Engineering Management, Liaoning Research Base of Equipment Manufacturing Development, Liaoning Key Research Base of Humanities and Social Sciences: Research Center of Micro-management Theory of SUT.

## Conflicts of interest/Competing interests

The authors declare no conflict of interest. We declare that there is no conflict of interests regarding the publication of this article.

## Availability of data and material

The data that support the findings of this study are available on request from the corresponding author.

## Ethics approval

This article does not contain any studies with human participants or animals performed by any of the authors. We claim that none of the material in the paper has been published or is under consideration for publication elsewhere.

## CRediT authorship contribution statement

**Jiating Liang:** Writing – original draft. **Peng Liu:** Writing – original draft, Methodology.

## Declaration of competing interest

The authors declare that they have no known competing financial interests or personal relationships that could have appeared to influence the work reported in this paper.
